# Fine Art in Dentistry

**Published:** 1872-08

**Authors:** 


### Fine Art in Dentistry.

BY W. F. M.

*Read Before the Indiana State Dental Association, June* 26, 1872.

In the rapid strides to high attainments in dental practice, the Fine Art has been slower in development than we could have desired. A limited number only of dentists practice their profession beyond mere gain in pocket. Those ideal perfections from which the esthetics are derived, claim none of their attentions. Study and cultivation of models both in art and nature, are rarely assiduously pursued by those who assume to practice dentistry. Our relations as art-students are too often imperfectly understood. Our early teachings and training have many times been crude and stinted. Nor were these early lessons but feebly impressed with anything like the refining attributes of to-day. Many of us do not realize that we are co-workers with painters and sculpturers, belonging to the great brotherhood of artists, and clothed with, perhaps, greater responsibilities.

Confined as our manipulations are to the human form or living face, we have the most pleasing object in nature—especially when it is upon a young and beautiful woman. With it we associate emotions of innocence, gaity, sensibility, intelligence, delicacy or vivacity. It is not dead matter with which we have to deal, but that which has feeling and sympathy of sentient beings. By disease, accident, congenital deformity, and shriveled age we are required to relieve, conserve, restore these human disfigurements, to add not only comfort and comeliness, but impart a symmetry and harmony which may have been lost, or which has never before existed. Our manipulations then must extend beyond the mere passable—must be faithful to nature and right reason. It is by a combination of such principles in representation or expression, given by the inventive faculty to certain lines, or other media, distributing agreeable proportions among the several parts as shall result in achievements, not only useful, but at the same time administer to our esthetical enjoyment.

Thus, in a cursory manner, I have endeavored to hint something of the nature of my subject, and now shall proceed to elucidate in detail the meaning of fine art in dental practice. It will likely occur to most of you who are painstaking, that a very large share of dental operations, presumed to come within your daily range of vision, evince no marks of beauty or taste whatever. In fact, to be more specific, they are often unsightly, shocking, destitute of every claim to even comeliness, and perhaps border upon the mere sham But of that class more like caricatures, chiefly from shops of stunning advertisers, whose wood cuts excel those of the jack or stallion, common about country stables, and which are so profuse in promise "to insure the foal," I shall not undertake to reckon within the pale of criticism. That commun ity which gives these *dental architects* encouraging support, is to be pitied rather than condemned. Of another and more respectible class, where some pretensions are regarded for the preservation of natural organs; but where large sacrifices of tooth substance were made, detrimental to the beauty as well as to the articulating surfaces, we have abundant proof that this class does not come within the scope of fine art.

The mouth viewed externally is, in point of attractiveness, next to the eye. It exhibits wonderful phases of character. In its changeful moods it is a powerful adjunct to all the passions—wreathing into smiles, or quivering with rage and scorn.

Our taste is improved by estimation of the degrees of excellence by comparison, proceeding from justice to refinement.

A standard type of manly beauty is the head of Apollo Belvidere, standing for centuries in the Vatican at Rome. Not many of us can afford to make a journey thither for the purpose of studying this model, which has stood the test of criticism so many ages. But from accurate drawings of this, and other models of great merit, we can learn very much with which to highten our ideal conception. And in proportion as our knowledge is increased in an esthetical point, in that ratio will we be enabled to achieve far higher and better results in dental practice.

In the profile of this head of Apollo you observe three exact divisions—one third forehead, one third nose, and one third lips and chin. No division can spare anything or add anything without marring the harmony of the entire features. There is almost a straight line from the roots of the hair on the forehead to the tip of the nose. The upper lip is of medium fullness, while the under curves and slightly inclines inward of the upper. A front view will disclose exact measurements from the corners of the mouth to the ears.

It is almost needless to inform you, that in artificial dentures, very limited indeed is the number of dentists who give heed and thorough attention to the external expression of the face, while engaged in this particular branch of their labors. Not a few of our professional brethren are unmindful of well-adapted plates, even if they wholly disregard proper expression of the features. We have for remedial aid in each case presented to us, such a one as will never be before us again, and our methods should be varied, changed so as to avoid giving a stiff, affected appearance. In short, conceal your art; and let there be a grace given it as shall neither fall short nor over-leap the demands of nature. Loss of teeth, absorption of the alveola borders, the wings of the nose drawn down, compressed lips, and the nose stooping to kiss the chin, are some of the disfigurements, which a dentist of true artist qualities about him may, in a great measure, restore or improve. But if we were to select an example on which superior skill must be exercised, and where nature can sometimes be improved, we have only to take one whose mouth has a short upper lip, a very projecting maxiliary, a pug nose, and a face thin. Or we may take one whose features are dark, shriveled, whose teeth have been lost many years, and whose inferior jaw projects beyond the upper, where your efforts to obtain articulation are somewhat baffled. Other cases still more suggestive, will readily occur to each of you, which present obstacles, even in the hands of the most experienced and skillful that are not easily overcome. If in the exercise of our skill on cases like these, we should happen to throw around them a "charm of beauty," you can readily surmise how the harmony would be destroyed. The mouth can be made too attractive. A most grievous fault is to have on exhibition, when a patient smiles, a row of twelve or fourteen teeth, with cuspids and bicuspids, having an undue prominence in the circle. We may copy or imitate nature, but do not in your dentures startle spectators with a ghastly grin, making them a centrality of sight. Be watchful that the cuspids have a proper position, not tiger-like, but that they give only the character needed. It will not escape your observation that in a large number of artificial dentures, the dividing line in the occlusion of the mouth, is not well taken; generally too short in distance, making the face appear when the teeth are brought together like some jack o'lantern. And in enunciation there are likewise egregious mistakes. A thickness of voice, a whistling accent that would indicate a fettered condition of movements of the mouth, occasioned by ill-constructed teeth, are too common for even the casual observers eye. Remember it is within your province to give each artificial denture just the required fullness and no more. To be too stinted, and have no difference in appearance of a patient after or just before, is about as palpable a mistake as any. Be particular in shades, color and sizes of your selections of teeth. Mark well your articulations; make the antagonizing points exact. This can only be done by letting the patient wear the denture for a few days, then place some plaster along the outside of the bicuspids remaining there until set, then remove, and get a new articulation. Examine carefully the antagonizing points; place wet paper between and draw out, letting the hitting points of the teeth tear away the paper. Grind away until your bearings be correct. Nor must you forget to give heed to the fly or loose muscles of the mouth, lying along the alveolar borders where the plates are apt to impinge and destroy much of the freedom of the mouth.

In the branch of operative dentistry, your daily observations are confirmed that, in the specialty of filling teeth there is a peculiar dearth in Fine Art. And how many are here to-day restoring teeth to their original shape, strength and preservation? Aye, let us whisper it, how many here to-day are not destroying equally as many teeth as they save? How many have got beyond a flat filling, or a tooth chiseled away until its walls look imploringly for the hand of an artist to bring adequate protection? Are they not expecting better things from us? Shall our journals and our preceptors expound to us their methods, pointing us the better way, and we still remain empty and vapid to all their guidances? Let us emancipate ourselves from these old, exploded practices, go up to a higher and more enlightened plain where the old masters have invited us. Let us make ourselves no longer the hewers of teeth, but the conservators of these organs. Not every dentist can be presumed to be endowed with all the great gifts of nature, or those that may be acquired; but by well directed industry, he may be able to cultivate those principles which underly the Fine Art as shall materially advance his professional accomplishments. The essence of the Fine Art in dentistry is not reached by inspiration alone. The human hand trained, educated, nerved, to respond quickly to that which the mind conceives, is alike essential. There is magic in the touch which only a practiced hand can give. But when guided by superior genius "the hand can not only round the dome of St. Peter's," *but fabricate precious jewels immortal in the mouths of Nations.*

